# 3T3 Cell Lines Stably Expressing Pax6 or Pax6(5a) – A New Tool Used for Identification of Common and Isoform Specific Target Genes

**DOI:** 10.1371/journal.pone.0031915

**Published:** 2012-02-23

**Authors:** Yury Kiselev, Tonje Engevik Eriksen, Siri Forsdahl, Lan Huong Thi Nguyen, Ingvild Mikkola

**Affiliations:** Research Group of Pharmacology, Department of Pharmacy, University of Tromsø, Tromsø, Norway; VIB & Katholieke Universiteit Leuven, Belgium

## Abstract

Pax6 and Pax6(5a) are two isoforms of the evolutionary conserved Pax6 gene often co-expressed in specific stochiometric relationship in the brain and the eye during development. The Pax6(5a) protein differs from Pax6 by having a 14 amino acid insert in the paired domain, causing the two proteins to have different DNA binding specificities. Difference in functions during development is proven by the fact that mutations in the 14 amino acid insertion for Pax6(5a) give a slightly different eye phenotype than the one described for Pax6. Whereas quite many Pax6 target genes have been published during the last years, few Pax6(5a) specific target genes have been reported on. However, target genes identified by Pax6 knockout studies can probably be Pax6(5a) targets as well, since this isoform also will be affected by the knockout. In order to identify new Pax6 target genes, and to try to distinguish between genes regulated by Pax6 and Pax6(5a), we generated FlpIn-3T3 cell lines stably expressing Pax6 or Pax6(5a). RNA was harvested from these cell lines and used in gene expression microarrays where we identified a number of genes differentially regulated by Pax6 and Pax6(5a). A majority of these were associated with the extracellular region. By qPCR we verified that *Ncam1*, *Ngef*, *Sphk1*, *Dkk3* and *Crtap* are Pax6(5a) specific target genes, while *Tgfbi*, *Vegfa*, *EphB2*, *Klk8* and *Edn1* were confirmed as Pax6 specific target genes. *Nbl1*, *Ngfb* and seven genes encoding different glycosyl transferases appeared to be regulated by both. Direct binding to the promoters of Crtap, Ctgf, Edn1, Dkk3, Pdgfb and Ngef was verified by ChIP. Furthermore, a change in morphology of the stably transfected Pax6 and Pax6(5a) cells was observed, and the Pax6 expressing cells were shown to have increased proliferation and migration capacities.

## Introduction

Pax6 is an evolutionary conserved transcription factor important for proper embryo development, while it also has crucial functions in certain tissues in adults. Pax6 is expressed in the central nervous system, the olfactory epithelium, the eye and the pancreas [1]. Heterozygous Pax6 mutations cause the eye phenotype aniridia in humans, and is also associated with glucose intolerance [2], lack of pineal gland and absence of the anterior comissure [3], as well as various neural phenotypes associated with aniridia [4] and references therein). Homozygous Pax6 mutants have no eyes, nasal structures and pancreas in addition to severe brain defects, and die shortly after birth [5]. Pax6 has two DNA binding domains: the bipartite paired domain (PD) and the paired type homeodomain (HD) [6]. Both can bind DNA independently, but they can also cooperate. The C-terminal part of Pax6 functions as a transcriptional activation domain [7], [8]. The Pax6ΔPD isoform contains only the HD and transcriptional activation domain (TAD). Moreover, a splice variant of Pax6 which has a 14 amino acid insertion in the N-terminal part of the PD gives rise to the Pax6(5a) isoform that has a completely different DNA binding specificity [9]. Isolated Pax6 DNA binding sites verified by DNA-footprinting are long (at least 20–30 bp) and seemingly versatile [10]–[15]. These sites represent Pax6 binding *in vivo* and can include contribution from both the paired domain and the homeodomain. A PCR based binding site selection method has been used to select the optimal binding sites for both the ordinary Pax6 paired domain [16] and the Pax6(5a) paired domain [9] *in vitro*. In this case the selected consensus sequences termed p6CON and 5aCON, were 20 and 22 bp long, respectively. Whereas p6CON is bound by one paired domain, the 5aCON consensus looks like two tandem repeats, and are preferably bound by four Pax6(5a) paired domains simultaneously [9], [17]. Importantly, Pax6(5a) is not able to bind P6CON sites, but Pax6 can bind 5aCON sites albeit as a monomer, and not as efficiently as Pax6(5a). 5aCON halfsites can be bound by Pax6(5a) monomers, but not by Pax6 at all [9].

The Pax6(5a) isoform is vertebrate specific [18], [19]. However, the eyegone (eyg) and twin-of-eyegone (toe) genes in Drosophila are thought to be Pax6(5a) homologues with regard to DNA binding properties since they lack the N-terminal part of the paired domain [20]. Both Pax6 isoforms are expressed together in various tissues in the eye and brain, and seem to functionally interact to stimulate transcription of target genes [21], [22]. In reporter gene assays the optimal ratio between Pax6 and Pax6(5a) was shown to be 8∶1 or 1∶1 [23]. In the early developing mouse brain the Pax6 and Pax6(5a) transcripts are expressed in a ratio of 8∶1 [17], [24] but from E12.5 to E14.4 this ratio falls to 3∶1 [24]. A decrease in the ratio between Pax6 and Pax6(5a) is also observed during chick retina development [25]. However in adult human lens and cornea, as well as in monkey retina, there seem to be equal levels of PAX6 and PAX6(5a) [26]. Even though they are expressed together, the two Pax6 isoforms have different functions. This is illustrated by the fact that mice lacking the Pax6-5a isoform have specific eye defects and also a difference in anatomy of the pancreas [27]. A missense mutation within Pax6 exon 5a has been described in four families with members suffering from Peters anomaly, congenital cataracts, Axenfeldt anomaly and/or foveal hypoplasia [28]. In line with this, in chicken retina the expression of Pax6(5a) is especially high in the fovea [25]. Pax6(5a) overexpression in transgenic mice causes cataracts and upregulation of alpha5-beta1-integrin [29]. Pax6 and Pax6(5a) are both reported to be involved in proliferation and differentiation in several studies, but they seem to play different parts. For example, *in ovo* electroporation of chick retina with either Pax6 or Pax6(5a) showed that both isoforms caused increased retinal cell proliferation, but Pax6(5a) induced ectopic differentiation of the retina to a stronger degree than Pax6 [25]. Pax6(5a) was also shown to strongly induce murine embryonic stem cells to differentiate into neurons, while Pax6 did not have such a strong effect [30]. However, Haubst and colleagues came to a different conclusion when they investigated the effects Pax6 mutations in the paired domain (PD), the PD-5a insertion and the homeodomain had on brain development [31]. They showed that Pax6 PD mutations affected both proliferation and differentiation, while the PD-5a mutation only affected proliferation and had no effect on cell fate/differentiation in brain development.

In the search for novel Pax6 target genes, gene expression microarrays have been used for both Pax6 overexpressing [21] and Pax6 heterozygous mutant mouse lenses [32], mouse Pax6 homozygous mutant forebrain [33], [34] and rat Pax6 homozygous mutant hindbrain [35]. There has also been performed a high-throughput screening using *in-situ* hybridization to look for genes regulated by Pax6 [36].

Not much is known about Pax6(5a) target genes, and the problem might be in the co-expression with Pax6 making it hard to distinguish between combined- and isoform-specific effects on gene expression. In this study we constructed Flp-In 3T3 cell lines stably expressing either Pax6 or Pax6(5a). 3T3-cells have no expression of the endogenous Pax6 gene locus and the constructed cell lines thus express only the stably transfected isoform, either Pax6 or Pax6(5a). They therefore seem to be ideal to study individual target genes regulated by either Pax6 or Pax6(5a). We have performed a gene expression microarray showing that majority of the regulated genes are associated with the extracellular region and include growth factors, growth factor receptors, cell adhesion molecules, extracellular matrix components, secreted inhibitors and peptidases. Interestingly, when target genes were analyzed with regard to gene ontology, the four groups of Pax6 and Pax6(5a) up- and downregulated genes had obvious differences in biological properties. A change in morphology of the Pax6 and Pax6(5a) expressing cells was also observed, as well as an increase in proliferation and migration.

## Results

### Constuction of Flp-In 3T3 cell lines expressing either Pax6 or Pax6(5a)

**Figure 1:**
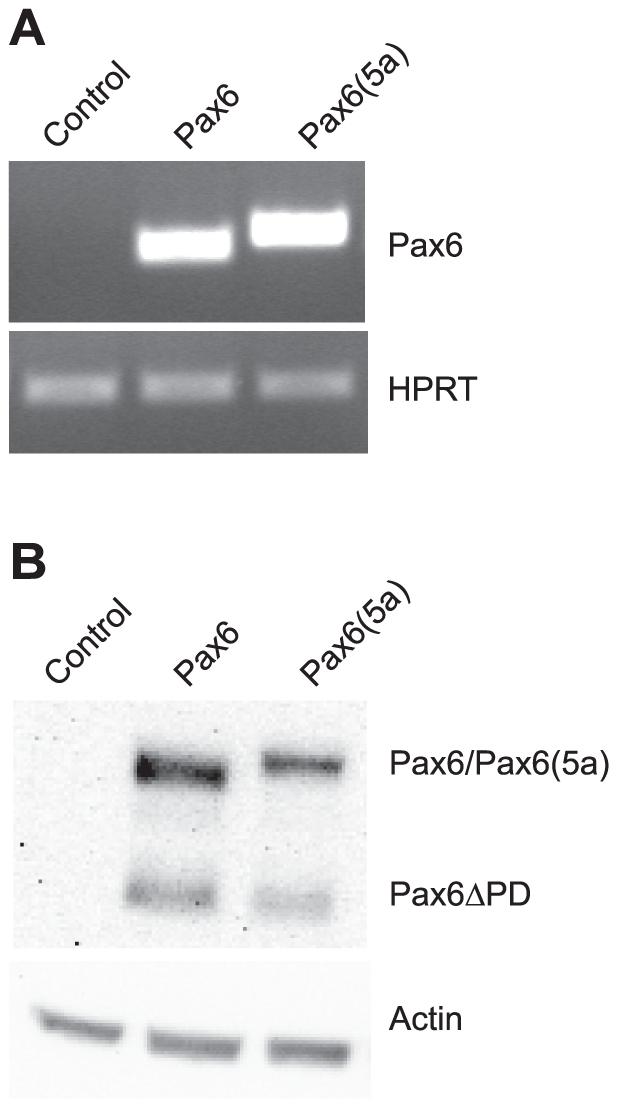
RT-PCR and Western blot confirm expression of the correct Pax6 isoform in the FlpIn-3T3 Pax6 and Pax6(5a) cell lines. (A) RT-PCR primers were designed so that the alternatively spliced exon 5a (42 bp) would be included in the PCR product if present. HPRT specific primers were used to show equal input of cDNA. Neither Pax6 nor Pax6(5a) is present in the FlpIn-3T3 control cell line. (B) Western blot with a Pax6 specific antibody (Millipore # AB2237) confirms expression of Pax6 and Pax6(5a) proteins in the respective cell lines. The lower molecular weight band of equal size for each of the Pax6 cell lines is the Pax6ΔPD isoform generated from an internal AUG startcodon in the link between the paired domain and the homeodomain.

The two Pax6 isoforms, *Pax6* and *Pax6(5a)*, are co-expressed in both the eye and the brain in tissue specific ratios. Searching for target genes by use of tissues or cell lines from *Pax6* mutant animals, or by knocking down *Pax6* with siRNA will not distinguish between Pax6 and Pax6(5a) target genes since both Pax6 isoforms will be affected. It could be that several of the identified Pax6 target genes are common for both isoforms, but one would expect each isoform to also be able also to regulate genes on their own since their binding site preferences are different. In order to identify new Pax6 target genes, and to try to distinguish between genes regulated by Pax6 and Pax6(5a), we generated FlpIn-3T3 (Invitrogen) cell lines stably expressing the mouse Pax6 or Pax6(5a) cDNA. The mouse fibroblast 3T3 cell line was specifically chosen because it does not express any endogenous *Pax6*. Successful flip-in and expression of Pax6 and Pax6(5a) were shown both by RT-PCR and Western blotting (Fig. 1 A and B).

### Gene expression microarray identifies both common and different sets of genes regulated by Pax6 and Pax6(5a)

**Figure 2:**
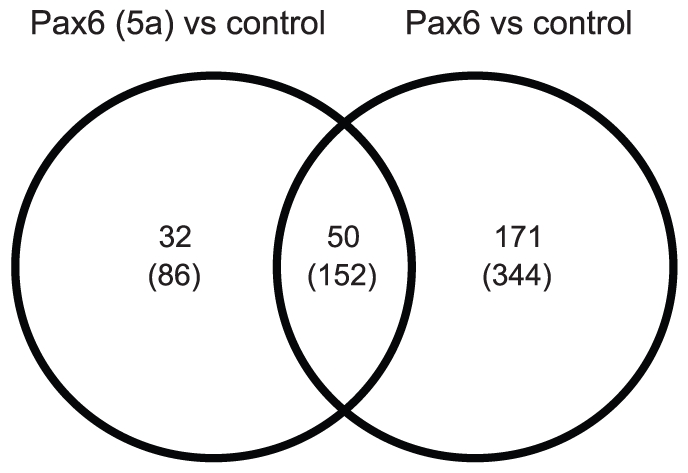
Venn diagram shows the number of individual and common sets of genes regulated by Pax6 and Pax6(5a) in a gene expression microarray. Three RNA samples from the FlpIn-3T3 control cell line, three samples from the FlpIn-3T3 Pax6(5a) cell line and two samples from the FlpIn-3T3 Pax6 cell line were sent for microarray analysis on the Illumina Mouse Ref-8 V1.1 chip. The number of genes differentially regulated as scored by threshold 1 is shown, while use of threshold 2 gave numbers shown in parentheses. (Threshold 1 is FDR p-value 0.001, FC 1.7, log FC 0.77. Threshold 2 is FDR p-value 0.01, FC 1.5, log FC 0.58).

Eight samples of RNA from the three different cell lines were sent for microarray analyses on an Illumina Mouse Ref-8 V1.1 chip (Turku Centre for Biotechnology, Finland). The samples included three different RNA preparations from two different passages for the FlpIn-3T3 (control) and FlpIn- Pax6(5a) cell lines, and two different RNA preparations from two different passages for FlpIn-Pax6 cell lines. We found a small but significant number of genes to be regulated by the two Pax6 isoforms when comparing gene expression in the 3T3-Pax6 and 3T3-Pax6(5a) cell lines to gene expression in the 3T3-FlpIn cell line. A total number of 221 genes were regulated by Pax6 (130 down and 81 up), whereas Pax6(5a) regulated a smaller number of genes, totally 82 (52 down and 30 up) with a false discovery rate (FDR/p-value) of <0.001 (Table 1). A Venn diagram (Fig. 2) shows that only 50 genes are regulated by both Pax6 and Pax6(5a), whereas Pax6 affects 171 genes and Pax6(5a) regulates 32 genes. The full list of genes regulated by Pax6 and by Pax6(5a) can be seen in Tables S1 and S2, respectively. Already known Pax6 target genes such as *Ncam1* [37], [38], *Vegfa* [39], *Dkk3* [34] and *Crabp1* [34] are on the list of regulated genes. Interestingly, *Ncam1* which is published as a Pax6 target gene, is only found on the Pax6(5a) list, where it is upregulated with a 2.4 fold change.

**Table 1:** Number of genes differentially regulated in the 3T3-Pax6 and 3T3-Pax6(5a) cell lines compared to the 3T3-control cell line.

	Pax6(5a)/control#	Pax6/control#	Pax6(5a)/control&	Pax6/control&
**Up**	30	81	108	207
**Down**	52	130	130	289
**Total**	82	221	238	496

# Threshold 1 (FDR p-value 0.001, FC 1.7, log FC 0.77),

& Threshold 2 (FDR p-value 0.01, FC 1.5, log FC 0.58), Illumina Mouse Ref-8 V1.1 chip.

### Gene ontology analysis shows that a majority of the regulated genes are associated with the membrane and extracellular matrix

The gene lists with up- or downregulated genes (threshold 1, see table 1) were analysed by the Database for Annotation, Visualisation and Integrated Discovery (DAVID) [40], [41].

To summarize, 25–30% of the genes regulated by both Pax6 and Pax6(5a) in the 3T3-FlpIn cell line are associated with the extracellular region when looking at the term cellular compartment (CC). Furthermore, looking at protein information resource (PIR) keywords, “glycoprotein”, “signal” and “secreted” are associated with 20–50% of the regulated genes in the Pax6 and Pax6(5a) cell lines (Table 2). All these observations fit with the molecular function (MF) of regulated genes being associated with growth factor binding, cytokine binding, carbohydrate binding, kinase- and receptor activities and enzymatic activities (Table 3). When one looks at the biological properties (BP) there are interesting differences between the four groups of genes: those associated with cell growth and adhesion are upregulated by Pax6, while genes associated with signalling pathways are downregulated. Pax6(5a) upregulated genes are associated with cell adhesion, motion and migration, but are also related to response to starvation and extracellular stimulus. Pax6(5a) downregulated genes are mostly associated with metabolic processes (Table 4).

**Table 2:** Percentage of Pax6/Pax6(5a) up- or downregulated genes associated with Gene ontology (GO) terms “Cellular compartment” and “SP_PIR_keywords”.

GO-term#	Pax6 up	Pax6 down	Pax6(5a) up	Pax6(5a) down
*Cellular compartment:*				
Extracellular region	25,00%	26,20%	32,10%	29,20%
Extracellular matrix	6,90%	10,30%	-	12,50%
Ancored to membrane	-	-	14,30%	-
Plasma membrane	-	31,70%	-	-
*SP_PIR_KEWORDS* $ *:*				
Secreted	22,00%	23,00%	32,10%	25,00%
Signal	30,60%	38,10%	46,40%	37,50%
Cell adhesion	9,70%	-	17,90%	-
Disulfid bond	25,00%	34,90%	42,90%	39,60%
Glycoprotein	27,80%	50,80%	39,30%	47,90%

# According to the Database for Annotation, Visualisation and Integrated Discovery (DAVID). Most significant (lowest p-value) at top.

$ SP_PIR = Swiss Protein_ Protein Information Resource.

**Table 3:** Percentage of Pax6/Pax6(5a) up- or downregulated genes associated with various Molecular Function (MF) gene ontology terms.

GO-term: MF#	Pax6 up	GO-term: MF#	Pax6 down	GO-term: MF#	Pax6(5a) up	GO-term: MF#	Pax6(5a) down
Insulin like growth factor binding	4,20%	Growth factor binding	5,60%	Growth factor activity	10,70%	Cytokine receptor activity	6,20%
Growth factor binding	4,20%	Polysaccharid binding	5,60%	Carbohydrate binding	10,70%	Cytokine binding	6,20%
Enzyme inhibitor activity	5,60%	Pattern binding	5,60%			Glucoronosyltransferase activity	4,20%
Serine type endopeptidase activity	4,20%	Cytokine binding	4,80%			Cytoskeletal protein binding	8,30%
Cytoskeletal protein binding	6,90%	Transmembrane receptor protein tyr kinase activity	4,00%			Endopeptidase activity	8,30%
Endopeptidase activity	6,90%	Vegf receptor activity	2,40%				
		Glucosaminoglycan binding	4,80%				
		Heparin binding	4,00%				

# According to the Database for Annotation, Visualisation and Integrated Discovery (DAVID). Most significant (lowest p-value) at top.

**Table 4:** Percentage of Pax6/Pax6(5a) up- or downregulated genes associated with various Biological Property (BP) gene ontology terms.

GO-term: BP#	Pax6 up	GO-term: BP#	Pax6 down	GO-term: BP#	Pax6(5a) up	GO-term: BP#	Pax6(5a) down
Regulation of cell growth	5,60%	Vegf signaling pathway	4,80%	Cell adhesion	17,90%	Uronic acid metabolic process	4,20%
Cell adhesion	11,10%	Enzyme linked receptor protein sign pathway	9,50%	Cell motion	14,30%	Glucoronate metabolic process	4,20%
Regulation of transmission of nerve impulses	5,60%	Transmembrane receptor prot tyr kinase sign pathway	7,90%	Cellular response to starvation	7,10%	Aminoglycan metabolic process	6,20%
Cell-cell adhesion	6,90%	Lung development	4,80%	Cell-cell adhesion	10,70%	Sulfur metabolic process	6,20%

# According to the Database for Annotation, Visualisation and Integrated Discovery (DAVID). The four most significant terms for each dataset are included, with the most significant (lowest p-value) at top.

Functional annotation clustering (which is a function in the DAVID database that cluster somewhat heterogeneous, yet highly similar, annotations into functional groups based on the degree of their co-associations with the genes on the users' individual gene lists), confirmed the above observations (data not shown). For both Pax6 and Pax6(5a) downregulated genes, the cluster giving the highest score contained the term ”extracellular matrix”. For Pax6 upregulated genes it was the cluster with the term “cell adhesion” that gave the highest score, while for Pax6(5a) upregulated genes the cluster with the highest score contained “disulfide bond” and “signal”, followed by a cluster containing the “cell adhesion” term.

### A group of glycosyl transferases are downregulated by Pax6 and Pax6(5a)

**Figure 3:**
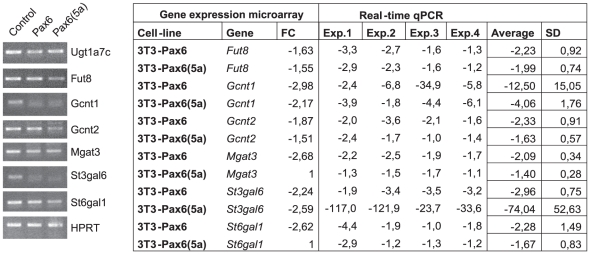
RT-PCR and real time qPCR with primers specific for seven glycosyl transferases confirms that this group of genes is downregulated by both Pax6 and Pax6(5a). Primers were designed against Ugt1a7, Fut8, Gcnt2, St3gal6, St6gal1, Gcnt1 and Mgat3. All showed downregulation of transcription of these genes in both the Pax6 and the Pax6(5a) cell lines compared to the FlpIn-3T3 control cell line. Pictures are representative of three experiments. The fold change value (FC) from the gene expression microarray is included in the table to the right. The Fut8 FC values for Pax6 and Pax6(5a), and the Gcnt2 value for Pax6(5a) have a p-value = 0,01(threshold 2), while the rest of the FC values listed have a p-value of 0.001 (threshold1). Four independent real-time qPCR experiments are included (Exp.1-Exp.4), and the results are presented as FC in cDNA concentrations in the FlpIn-3T3 Pax6 and Pax6(5a) cell lines compared to the FlpIn-3T3 control cell line, after normalization against two housekeeping genes (Nono and Tfrc).

An additional gene ontology database (eGOn, http://www.genetools.microarray.ntnu.no/adb/index.php) was also used for analysis [42]. With this tool one can compare lists of genes and look for similarities or differences. We compared the list of upregulated genes with the list of downregulated genes for both Pax6 and Pax6(5a), with the purpose to see if there were significant differences with regard to molecular function or biological properties in the groups of genes being up- or downregulated. The result showed that a significant group of genes being downregulated by Pax6 and Pax6(5a) is glycosyl transferases, while significantly more genes associated with the term kinase where found in the upregulated gene lists. This would imply that changed expression of *Pax6* and/or *Pax6(5a)* could cause post-translational differences in both the glycosylation pattern and phosphorylation status of proteins. This will probably have impact on cell adhesion, motility and signalling pathways. RT-PCR verified that all the tested glycosyl transferases (*St6gal1*, *St3gal6*, *Fut8*, *Mgat3*, *Gcnt1*, *Gcnt2* and *Ugt1a7c*) were downregulated in the *Pax6* and *Pax6(5a)* expressing FlpIn-3T3 cells (Fig. 3). qPCR confirmed the RT-PCR results, but the fold magnitude of downregulation varied between experiments. Therefore the result of four independent qPCRs are shown in the figure 3. For most of the glycosyl transferases transcripts, a modest (two-fold) downregulation was observed. However, Gcnt1 and particularly St3gal6 showed strong downregulation by Pax6 and Pax6(5a) respectively. The high deviation in fold downregulation could be caused by the sensitivity of the qPCR method which enable us to detect very small amounts of transcripts (giving high Ct values, with high deviation). Alternatively, other factors than Pax6(5a) may also influence the expression of St3gal6 contributing to the observed variation in expression levels.

### Pax6 and Pax6(5a)specific target genes are verified by qPCR

**Figure 4:**
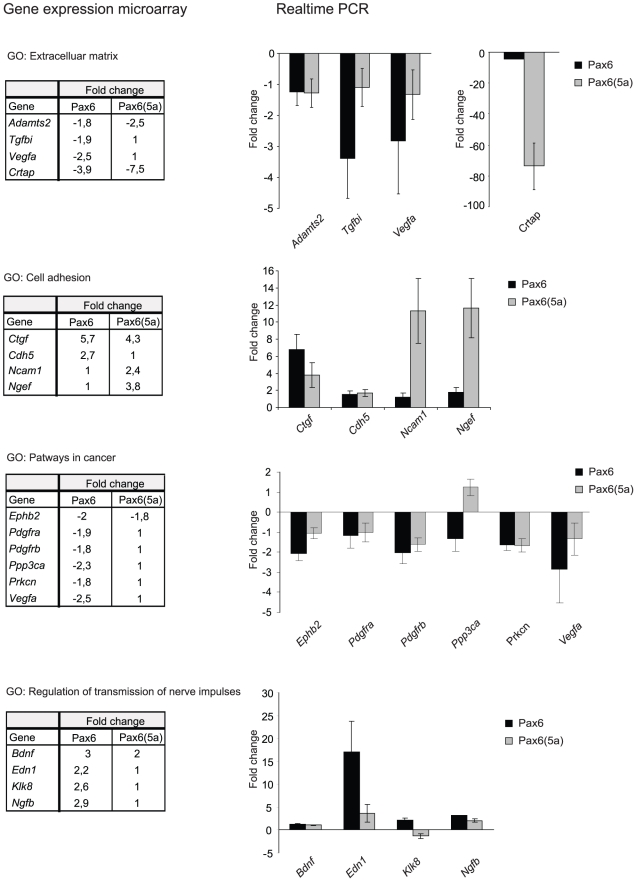
Real time quantitative PCR verifies differentially regulated genes in both the FlpIn-3T3 Pax6 and Pax6(5a) cell lines. Primers were designed for genes representative for the gene ontology terms “Extracellular matrix” (Adamts2, Tgfbi, Vegfa and Crtap), “Cell adhesion” (Ctgf, Cdh5, Ncam1 and Ngef), “Pathways in cancer” (Ephb2, Pdgfra, Pdgfrb, Ppp3ca, Prkcn and Vegfa) and “Regulation of transmission of nerve impulses” (Bdnf, Edn1, Klk8 and Ngfb) and used in qPCR. Results are means of three independent experiments and are presented as fold change in cDNA concentrations in the FlpIn-3T3 Pax6 and Pax6(5a) cell lines compared to the FlpIn-3T3 control cell line, after normalization against two housekeeping genes. The fold change values obtained for the same genes in the Illumina gene expression microarray are given in the table to the left of each graph for comparison.

Quantitative real time PCR primers were designed for some of the genes associated with extracellular matrix (*Tgfbi*, *Vegfa*, *Adamts-2 and Crtap*) and cell adhesion (*Ctgf*, *Cdh5 and Ncam*). Some genes representing the KEGG pathway “Pathways in cancer” (*Pdgfra*, *Pdgfrb*, *Vegfa*, *PPP3ca*, *EphB2 and Prkcn*) and the gene ontology term “regulation of transmission of nerve impulses” (*Bdnf*, *Ngfb*, *Klk8 and Edn1*) were also included (Fig. 4). As well, we performed qPCR on some genes listed as Pax6(5a) specific target genes (*Ncam1*, *Pdgfb*, *Ngef*, *Dkk3*, *Hist1h1c*, *Sphk1*and *Nbl1*). When a cut-off of fold change (FC) 1.7 was used, most of the genes showed up- or downregulation as expected from the microarray result. However, *Adamts2*, *Pdgfra*, *Ppp3ca* and *Bdnf* did not seem to be regulated at all, and *Prkcn* value was borderline. According to microarray results *Tgfbi*, *Vegfa*, *EphB2*, *Klk8* were expected to be regulated by Pax6 only, and this was confirmed by qPCR. Likewise, *Crtap* was downregulated and *Ctgf* was upregulated as expected by both Pax6 and Pax6(5a). We noticed that *Crtap* is much stronger downregulated by Pax6(5a) (74 fold) than by Pax6 (4 fold). Further, for several of the Pax6 specific target genes tested, real time PCR showed regulation by the Pax6(5a) isoform as well (e.g *Edn1*, *Ngfb*, *Cdh5*), even though the *Edn1* gene in particular seemed to be much stronger regulated by Pax6 than Pax6(5a) (17 fold compared to 3.7 fold) (Fig. 4).

### Real time qPCR confirms Ncam1, Ngef, Dkk3 and Sphk1 as Pax6(5a) specific targets

**Figure 5:**
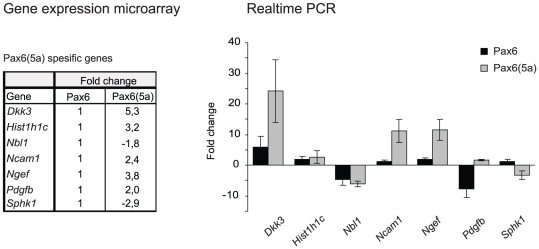
Real time quantitative PCR shows that Dkk3, Ncam1, Ngef and Sphk1 are strongly regulated by Pax6(5a). Primers were designed for some of the genes showing differential expression only in the FlpIn-3T3 Pax6(5a) cell line (Dkk3, Hist1h1c, Nbl1, Ncam1, Ngef, Pdgfb, Sphk1). Real time quantitative PCR was performed as described in Fig. 4. The fold change values obtained for the same genes in the Illumina gene expression microarray are given in the table to the left of the graph for comparison.

Generation of the Pax6(5a) expressing FlpIN-3T3 cell line provided an unique opportunity to search for genes specifically regulated by Pax6(5a). As the Venn diagram in figure 2 implies, 82 genes are indicated as targets for Pax6(5a), with 50 of these also being regulated by Pax6, leaving 32 as exclusive Pax6(5a) targets (Table 5). Quantitative real time PCR was performed with primers designed for some of the exclusive Pax6(5a) target genes listed in table 5: *Ncam1*, *Pdgfb*, *Ngef*, *Dkk3* and *Hist1h1c* (upregulated) and *Sphk1*and *Nbl1* (downregulated). The overall result confirmed the gene expression microarray data, though there were differences in the fold regulation compared to the microarray. However, most of the genes seemed to be regulated by Pax6 in addition to Pax6(5a), even though Pax6(5a) regulation was more prominent (Fig. 5). *Ncam1* has already been identified as a Pax6 target gene [37], [38]. Interestingly, the FlpIn 3T3-Pax6 cell line does not have significant increase in transcription of *Ncam1* compared to control cells, while the qPCR results from the Pax6(5a) cell line show that it is upregulated 11 fold (Fig. 5). Expression of *Ngef* is about 12 fold upregulated in the Pax6(5a) cell line. *Dkk3* is 24 fold upregulated in the Pax6(5a) cell line, and almost 6 fold upregulated in the Pax6 expressing cell line. *Sphk1* was differentially regulated only in the Pax6(5a) cell line (about 3 fold down), while *Nbl1*, which was also supposed to be a Pax6(5a) specific target gene, was almost equally downregulated by Pax6 and Pax6(5a) (−4.7 and −6.0 fold, respectively, Fig. 5) in the qPCR assay. Of notice, Pdgfb which was identified by the microarray as an exclusive Pax6(5a) target gene, showed only a slight upregulation by Pax6(5a) in qPCR (1.6 fold). However, Pdgfb turned out to be −7.8 fold downregulated in the 3T3-Pax6 cell line. It is an interesting deviation from the microarray results which we have no explanation for.

**Table 5:** Unique Pax6(5a) regulated genes.

Entrez_ID	Symbol	Gene name	FC
229672	Gm566	NA	7,859115
50781	Dkk3	dickkopf homolog 3 (Xenopus laevis)	5,279555
53972	Ngef	neuronal guanine nucleotide exchange factor	3,776922
50708	Hist1h1c	histone cluster 1, H1c	3,248262
68024	Hist1h2bc	histone cluster 1, H2bc	2,74685
17069	Ly6e	lymphocyte antigen 6 complex, locus E	2,735913
57875	Angptl4	angiopoietin-like 4	2,485674
17967	Ncam1	neural cell adhesion molecule 1	2,44828
192897	Itgb4	NA	2,296179
213948	Atg9b	ATG9 autophagy related 9 homolog B (S. cerevisiae)	2,178804
18591	Pdgfb	platelet derived growth factor, B polypeptide	2,002118
76071	Jakmip1	janus kinase and microtubule interacting protein 1	1,945522
21873	Tjp2	tight junction protein 2	1,883161
69550	Bst2	bone marrow stromal cell antigen 2	1,799411
225608	Sh3tc2	SH3 domain and tetratricopeptide repeats 2	1,787549
69032	Lyzl4	lysozyme-like 4	1,739619
620078	LOC620078	RIKEN cDNA C130026I21 gene	1,73713
17064	Cd93	CD93 antigen	1,726663
110074	Dut	deoxyuridine triphosphatase	−1,80608
17965	Nbl1	neuroblastoma, suppression of tumorigenicity 1	−1,81722
15893	Ica1	islet cell autoantigen 1	−1,90903
100689	Spon2	spondin 2, extracellular matrix protein	−1,96962
17022	Lum	lumican	−1,99828
21961	Tns1	NA	−2,00857
223332	Ranbp3l	RAN binding protein 3-like	−2,09339
12061	Bdkrb1	bradykinin receptor, beta 1	−2,1142
16164	Il13ra1	interleukin 13 receptor, alpha 1	−2,11692
331532	Tceal5	transcription elongation factor A (SII)-like 5	−2,14302
13179	Dcn	decorin	−2,32046
216725	Adamts2	a disintegrin-like and metallopeptidase (reprolysin type) with thrombospondin type 1 motif, 2	−2,52677
20698	Sphk1	sphingosine kinase 1	−2,93929
319909	5430433G21Rik	RIKEN cDNA 5430433G21 gene	−3,08482

### Bioinformatic analysis indicates Pax6 and Pax6(5a) binding sites in selected target genes

Both Pax6 and Pax6(5a) have very divergent consensus binding sites around 20 nucleotides long. For Pax6, the consensus site selected by *in vitro* binding of recombinant Pax6 to random oligonucletotides, SELEX [16] and the consensus site selected based on known Pax binding sites [6] were both used to search for Pax6 binding sites in the promoter regions of some of the selected target genes. There are not many published Pax(5a) target genes, so not a lot of *in vivo* binding sites have been identified. However, one study describing *in vitro* selection of binding sites for this isoform has been published [9]. The three different consensus sites p6CON [16], Pax6 consensus [6] and 5aCON half-site [9] were used to search for patterns in the promoters of mouse *Dkk3*, *Ncam1*, *Ngef*, *Sphk1*, *Ctgf*, *Edn1*, *Crtap*, *Vegfa* and *Pdgfb* by the use of GCG Findpatterns, allowing 2 mismatches. Two thousand bp upstream and 500 bp downstream of the first exon were used as input for each gene. The two used Pax6 binding sites gave several hits for each promoter only when a mismatch of 2 nucleotides was allowed (data not showed). Given that the Pax6 consensus sequence is quite degenerate to start with, the value of the obtained sites with two allowed mismatches is not clear. More attention was paid to the possible Pax6(5a) binding sites, since less is known about Pax6(5a) target genes in the literature. Results for the 5aCON half-sites are summarized in Table S3. For *Dkk3* several possible Pax6 sites (p6CON) are located in the promoter region, while only one Pax6(5a) (5aCON) half-site is located 200 bp downstream of the TSS in exon1. This site is conserved in the human *DKK3* gene as well (data not shown). *Ncam1* and *Ngef*, which also are more strongly regulated by Pax6(5a) than by Pax6, have several 5aCON half-sites located in their promoter regions. Ncam1 and Ngef have transcripts starting from two different regions according to the Ensembl genome database, but the promoter regions of transcripts for both genes (as defined above) have several 5aCON half-sites. For Ngef it is of interest to note that all four 5aCON half-sites are located downstream of the TSS for transcript 202, with three of them clustered around position −300 bp. For Ncam the 5aCON half-sites are located both upstream and downstream of the TSS. The Ctgf promoter has five 5aCON half-sites, all upstream of the TSS. No consensus 5aCON half-sites were found for the Sphk1, Vegfa, Pdgfb and Crtap promoters.

If Pax6 regulation of the target genes is important one would expect evolutionary conservation of Pax6 binding sites in their regulatory regions. We used an area of 5000 bp upstream and 1000 bp downstream of the TSS of selected target genes to check for conserved sites between mice and humans in rVista. Chicken and zebrafish were also included if the corresponding promoter areas of the genes were identified. The analyses were done for Dkk3, Ctgf, Crtap, Pdgfb, Edn1 and St3gal6. We intended to include Ncam1, Gcnt1 and Gapdh as well, but here the mouse and human promoters were not very much conserved to start with, so we did not continue. The other mouse-human promoter pairs were comparable with at least some degree of conservation, particularly in the area of the TSS. rVista uses transcription factor binding sites from the Transfac database. Identified Pax6 binding sites conserved between mouse and human promoters are shown in table 6. For the 6000 bp area covering the TSS there were multiple predicted Pax6 binding sites conserved between mouse and human: Ctgf (39), Edn1 (21), Pdgfb (18), Crtap (8), St3gal6 (8) and Dkk3 (2). Among the 39 Pax6 binding sites conserved between mouse and human for the Ctgf gene, one was also conserved in chicken, one in zebrafish, and one site was conserved in all four species. Among the 8 Pax6 binding sites conserved between human and mouse for the Crtap gene, 4 were also conserved in chicken. Thus there seem to exist evolutionary conserved Pax6 binding sites in the regulatory regions of several of the identified target genes indicating that their expression indeed can be directly regulated by Pax6.

**Table 6:** Evolutionary conserved Pax6 binding sites identified in the promoter region of several target genes using rVista.

Gene	Species	Transcript (Ensembl)	Region −5000/+1000 in the promoter region relative to the TSS (Ensembl)	# of evolutionary conserved Pax6 bs (rVista)
Dkk3	mouse	201	Chr7: 119301572–119307571	2
	human	202	Chr11: 12029178–12035177	
Crtap	mouse	001	Chr9: 114298794–114304793	8
	human	001	Chr3: 33150471–33156470	
Ctgf	mouse	001	Chr10: 24310248–24316247	39
	human	001	Chr6: 132271514–132277513	
Edn1	mouse	201	Chr13: 42391639–42397638	21
	human	001	Chr6: 12285596–12291595	
Pdgfb	mouse	201	Chr15:79844239–79850238	18
	human	001	Chr22: 39639757–39645756	
St3gal6	mouse	001	Chr16: 58522540–58528539	8
	human	001	Chr3: 98446099–98452098	
Crtap	chicken	-	Chr2: 44496250–44502249	4#
Ctgf	chicken	-	Chr3: 59158394–59164393	2$
Ctgf	zebrafish	001	Chr20: 25365421–25371420	2*

# Four of the eight sites evolutionary conserved between mouse and human are also conserved in chicken Crtap.

$ Two of the 39 sites conserved between mouse and human Ctgf gene are also conserved in chicken, one of these two sites is in addition conserved in zebrafish.

*Two of the 39 sites conserved between mouse and human Ctgf gene are also conserved in zebrafish, one of these two sites is in addition conserved in chicken.

### ChIP-PCR verifies binding of Pax6 and Pax6(5a) to the promoters of Crtap, Ctgf, Edn1, Pdgfb, Dkk3 and Ngef

**Figure 6:**
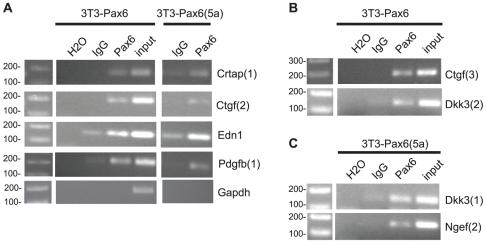
Chromatin immunoprecipitation verifies binding of Pax6 and Pax6(5a) to the promoter region of several target genes. Chromatin immunoprecipitation with an antibody against Pax6 was done for both the 3T3-Pax6 and the 3T3-Pax6(5a) cell line. IgG was used as a control. PCR primers were designed so that they should amplify target sequences with putative Pax6 and/or Pax6(5a) binding sites in the promoter regions of Crtap, Ctgf, Edn1, Pdgf, Dkk3 and Ngef. The number in parenthesis specifies the identity of the primerset used for the observed amplification. Genomic DNA sampled before adding the antibody (input) was used as a positive control for the PCR reaction, while water was used as a negative control. The promoter region of Gapdh was included as a region not expected to bind Pax6 or Pax6(5a).

Based on the bioinformatic analyses PCR primers were designed for use in ChIP experiments. Primer sets covered Pax6 and Pax6(5a) putative binding sites, and were used for PCR with ChIPed DNA from both 3T3-Pax6 and 3T3-Pax6(5a) cell lines. Some of the primer sets showed enrichment (binding) in both cell lines, indicating that both Pax6 and Pax6(5a) were associated with the promoters. This was observed for Crtap, Ctgf, Edn1, and Pdgfb (Fig. 6A). As expected, the Ngef promoter fragment containing the putative Pax6(5a) binding site was only enriched in immunoprecipitated chromatin from the 3T3-Pax6(5a) cell line (Fig. 6C), supporting our gene expression- and qPCR-results indicating that this is a true Pax6(5a) target gene. Importantly, no amplification was detected with either the Pax6 or Pax6(5a) ChIP extracts with primers directed against the promoter region of the housekeeping gene Gapdh which is not expected to bind Pax6 (Fig. 6A). Presence of faint bands in the IgG sample for some of the primer sets in figure 6 indicates unspecific binding of chromatin to either IgG or the protein A agarose beads. Since the PCR products of the Pax6 ChIPed samples are much more abundant (observed repeatedly) we feel confident that this area of the promoter is enriched in the Pax6 ChIPed samples. It should be noted that PCRs with two primer sets for Ncam1, one for Vegfa, and a second primer set for Ngef did not give any amplification in ChIPed extracts, indicating that the primers did not cover the Pax6 binding regions of these promoters (data not shown).To conclude, binding of Pax6 and/or Pax6(5a) to the promoters of several of the genes differentially expressed according to the gene expression microarray indicates that these are direct target genes of Pax6 and Pax6(5a).

### The morphology of Pax6 and Pax6(5a) FlpIn-3T3 cell lines is changed along with the proliferation rate and migration capacity compared to the original FlpIn 3T3 cell line

**Figure 7:**
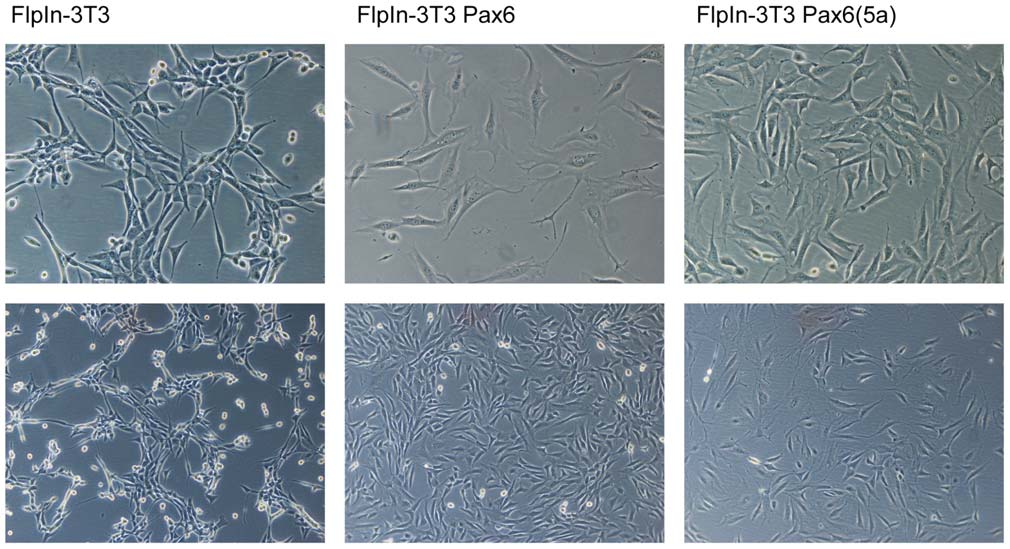
The FlpIn-3T3 Pax6 and FlpIn-3T3 Pax6(5a) cell lines have changed morphology compared to the original FlpIn 3T3 cell line they were generated from. This was repeatedly observed over several passages. Pax6 and Pax6(5a) expressing cells appeared more flattened and seemed to have more protrusions. The cells on the picture have passage numbers 17, 15 and 11 for the FlpIn-3T3, FlpIn-3T3 Pax6 and FlpIn-3T3 Pax6(5a) respectively. For the pictures on the upper row a 20× magnification is used, for the lower row a 10× magnification is used on a Zeizz Axiovert S100 Microscope with a Nikon Digital Sight DS-5 M camera.

**Figure 8:**
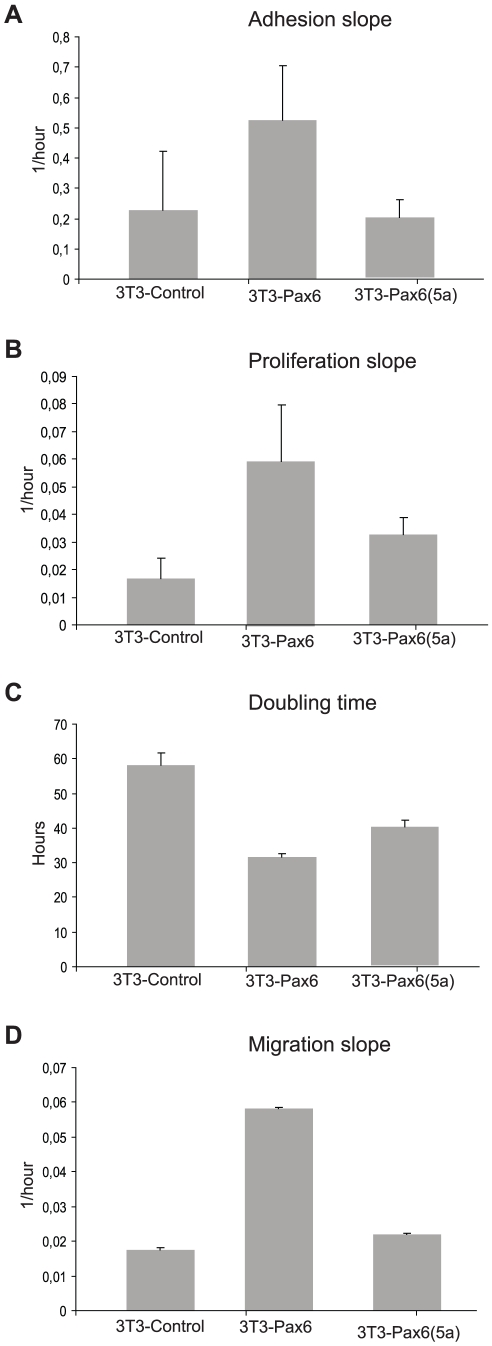
Pax6 expressing FlpIn-3T3 cells have higher proliferation and higher migration capacity compared to FlpIn-3T3 control cells and Pax6(5a) expressing cells. Control, Pax6 and Pax6(5a) cells were seeded in xCELLigence (Roche) proliferation plates, cells were left on the bench for 1 hr before the plates were put in the xCELLigence machine for measurements for 24 hrs. The measured values were used to calculate the (A) adhesion slope, (B) proliferation slope and (C) doubling time. For adhesion and proliferation slope analyses the mean and standard deviation of three independent experiments are shown. The doubling time analysis shows one representative experiment out of three, because absolute values were changing from experiment to experiment probably due to fluctuations in cell condition. However, described pattern of increased proliferation (Pax6>Pax6(5a)>control) was consistent in all experiments. Each experiment was done in triplicates (A–C). xCELLigence migration plates were used to measure the migration, and to calculate the migration slope (D). One representative experiment out of 3 is shown.

Consistent with the fact that about half of the target genes identified by the gene expression microarray are associated with the membrane and extracellular region, there is a change in morphology from the FlpIn-3T3 parent cell line to the Pax6 and Pax6(5a) cell lines. The Pax6 and Pax6(5a) cells are bigger and more flattened, and seemingly have more protrusions (Fig. 7). In order to evaluate if Pax6 or Pax6(5a) can influence major biological processes in 3T3 cells, we employed a recently introduced xCELLigence platform (Roche) which facilitates studying of cell proliferation and migration in real time. This is a microelectric assay based on changing impedance of electrodes in presence of cells. We recognize that differences in morphology of the three types of 3T3 cells studied (control, Pax6 and Pax6(5a)) could make impedance-derived absolute values misleading. Therefore the data are presented as curve slopes or doubling times. Attachment and initial spreading of the cells typically took 3±1 hours, and this time interval was chosen for calculation of attachment slopes as depicted on figure 8A. We observed repeatedly that 3T3-Pax6 cells attach more rapidly than 3T3-control cells, while no or little difference was seen for 3T3-Pax6(5a) cells compared to the control. In all experiments (n = 4) the 3T3 cell lines expressing either of isoforms of Pax6 had higher proliferation rates compared to the control cells, with 3T3-Pax6 being most prominent. This is shown both by proliferation slope analysis (Fig. 8B) and doubling time calculation (Fig. 8C). Finally, we studied whether serum starved 3T3-Pax6 or 3T3-Pax(5a) cells had altered migration capabilities in presence of a serum attractant compared to the 3T3-control cells. 3T3-control cells were unable to migrate through the uncoated xCELLigence CIM-plate membrane in any of the three performed experiments. To minimize the possibility that the observed difference in migration could be due to faster proliferating Pax6 cells the experiment was limited to 24 hrs. Furtermore, cells attached to the membrane at the end of the experiment were stained, and confirmed several fold differences in cell numbers (data not shown), ruling out the possibility that the observed changes in impedance were solely caused by adherence differences. Interestingly, Pax6 expressing cells did actively pass through the membrane pores following the serum gradient, while Pax6(5a) seemed to have very limited influence on the migration abilities of the 3T3 cells (Fig. 8D).

## Discussion

In this study FlpIn-3T3 cell lines expressing either *Pax6* or *Pax6(5a)* were generated and used to search for target genes. Gene ontology analysis of the resulting gene expression microarray indicated that a substantial amount of both up- and downregulated genes was associated with the extracellular region and cell membrane. Regulated genes were also associated with keywords such as “glycoprotein”, “secreted” and “signal”. There are differences in the group of genes regulated by Pax6 and Pax6(5a), with Pax6 upregulated genes being associated with cell growth and adhesion, and Pax6(5a) upregulated genes being associated with cell motion and migration, as well as response to extracellular signaling and starvation. Downregulated genes were associated with receptor protein signaling pathways for Pax6 and several metabolic processes for Pax6(5a). Selected genes were verified as targets for Pax6 and/or Pax6(5a) by use of qPCR and ChIP. The possible biological meaning of these results will be discussed below. Most interestingly, several Pax6(5a) specific target genes were identified.

### Relevance of the cell line used

There is no endogenous *Pax6* expression in the murine 3T3 fibroblast cell line. The FlpIn-3T3 cells were therefore chosen to create a Pax6 and a Pax6(5a) cell line to avoid background expression when performing the gene expression microarray. We are aware that certain co-factors that co-regulate transcription by Pax6 might be missing in the 3T3 cell line, and that this most probably influenced the resulting number of differentially regulated genes. In addition, since Pax6 and Pax6(5a) also are normally co-expressed, they probably influence the transcriptional activation/repression activity and promoter specificity of each other. However, these disadvantages can be overcome in future by transfecting these cell lines with the appropriate co-factors, or the missing Pax6 isoform, to look at the effect this will have on target gene expression. The already known Pax6 target genes such as *Ncam1* [37], [38], *Vegfa* [39], *Dkk3* [34] and *Crabp1* [34] are also present on the list of regulated genes in our experiment. This supports the validity of these cell lines for target genes search, and strengthens our finding of novel Pax6 and Pax6(5a) target genes.

It has been reported that Pax6 and Pax6(5a) positively autoregulate expression from the endogenous *Pax6* promoter in Neuro2D and NIH3T3 cell lines [43], but we did not observe this throughout the lengthy study in the FlpIn-3T3 cell lines. The FlpIn-3T3 Pax6 cell line never expressed *Pax6(5a)*, and the FlpIn-3T3 Pax6(5a) cell line did not turn on the endogenous *Pax6* gene. A possible explanation for this could be that the chromatin packing in the FlpIn-3T3 cells is such that the *Pax6* autoregulatory binding sites are not available, or some co-factors are not present The discrepancy between our results and the result of Pinson et al 2006 may be caused by subtle differences between the chromatin packing in NIH3T3 and FlpIn-3T3 cells. The transfection methods or the insertion sites of the FlpIn cassette could have also contributed to the observed differences.

Expression of neuron-specific genes upon transduction with *Pax6* has previously been shown in HeLa cells [44]. Similar to our observations in the 3T3 fibroblasts, Cartier and co-workers showed that HeLa cells change morphology and start to migrate upon stable transfection with *Pax6* [44]. However, although we also observed expression of some genes with known functions in neurons (*Ngef*, *Ngfb*,*Edn1 EphB2*, *EphB4*, *Klk8*), we did not observe a post-mitotic phenotype. On the contrary, Pax6 cells seemed to increase their proliferation rate compared to the control 3T3-FlpIn cells. Interestingly, whereas Pax6(5a) also seemed to have a positive effect on the proliferation of the FlpIn-3T3 cells, it appearently did not increase migration, indicating a functional difference in the genes regulated by Pax6 and Pax6(5a). However, there are no reports on Pax6(5a) being expressed alone *in vivo*; it is always reported to be present in combination with Pax6 in different ratios. Since Pax6 and Pax6(5a) obviously have different sets of target genes, one might expect that a change in the Pax6∶Pax6(5a) ratio would cause a change in the gene expression pattern, ultimately changing the cell properties.

### Possible function of Pax6 regulation of target genes expressed in the extracellular region

In this study we have shown that stable expression of *Pax6* or *Pax6(5a)* in a fibroblast cell line causes changes in expression of genes associated with the extracellular region and the membrane. These genes include receptors, adhesion molecules, secreted growth factors, secreted inhibitory molecules and matrix modifying enzymes. In addition, several glycosyl transferases are downregulated, so the glycosylation pattern on the molecules expressed on the outside of the cells is also expected to be different compared to fibroblasts not expressing *Pax6*. Some of the target genes identified by this approach are already reported to be Pax6 target genes identified in brain, such as *Ncam1*, *Vegfa*, *Dkk3*, *S100A11* and *Crabp1* [34], [38], [39]. Further, the seemingly high proportion of Pax6 regulated genes in the extracellular region in this fibroblast cell line (secreted or associated with the membrane), is supported by several lines of evidence in the literature from more biologically relevant cells and tissues. Particularly in the brain, *Pax6*-positive cells are reported to express specific cell surface molecules or secret specific extracellular matrix components or modifying enzymes. For example, later born cortical precursor cells in homozygous *Pax6* mutant mouse fail to leave the subventricular zone (SVZ). When transplanted into wild-type cortex they migrate and differentiate normally, showing that the inability to exit from the SVZ is non-autonomous, and that *Pax6* is needed to generate a cortical environment that permits the later born precursors to migrate and differentiate [45]. Similarly, *Pax6* is expressed in the anti-hem region shown to be a signaling centre in the brain. In *Pax6* knockout mice cells normally found on the ventral side migrate to the dorsal side, indicating that *Pax6* expressing cells in the antihem function as a barrier, separating the dorsal and ventral telencephalon [46]. Neural crest derived stem cells (NCDD) play a crucial role in the development of the front-nasal tissue. These cells migrate from the midbrain, but in *Pax6* homozygous mutant embryos they cannot invade the front nasal mass [47], due to non cell-autonomous defects in migration. The HNK-1 epitope is known to inhibit NCDD migration, and this epitope is ectopically expressed in the *Pax6* mutant rat facial ectoderm [48]. It could be that *Pax6* expression normally is needed to repress the expression of HNK-1 epitope to allow passage of NCDDs on their way to the front-nasal area. Pax6 is also shown to regulate migration of neuronal precursors in the cerebral cortex, and the authors suggested that the primary role of Pax6 during cortex development was to regulate cell surface properties responsible for radial migration and cellular identity [49].

### Pax6(5a) target genes

For this study it was absolutely necessary to have a separate cell line for each Pax6 isoform, to be able to distinguish target genes that are specific to, or common for Pax6 and Pax6(5a). We identified 32 exclusive Pax6(5a) target genes in the 3T3-Pax6(5a) cell line. Of the ones tested in qPCR, *Dkk3*, *Ngef* and *Ncam1* showed the highest fold upregulation by Pax6(5a) (approximately 24, 12 and 11 fold, respectively). However, qPCR showed that *Dkk3* and *Ngef* were also regulated by Pax6, but to a much lesser extent – about 2 fold for *Ngef* and 6 fold for *Dkk3*. When analyzing the promoters of these genes (2000 bp upstream and 500–600 bp downstream of the transcriptional start site) in GCG Findpatterns, several putative Pax6 and Pax6(5a) binding sites were found when up to two mismatches compared to the P6CON and 5aCON sites were allowed [9], [16]. Interestingly, *Dkk3* was the only gene containing a sequence similar to the tandemly arranged Pax6(5a) consensus site (5aCON, [9]), indicating that binding of four Pax6(5a) paired domains could be possible also on an *in vivo* regulated target gene.

Dkk3 is a member of the Dickkopf (Dkk) family of secreted glycoproteins which is a major class of Wnt signaling regulators, typically antagonizing Wnt/β-catenin signaling (reviewed in [50]). While Dkk1, Dkk2 and Dkk4 inhibit Wnt signaling by binding to LRP5/6 preventing Wnt and Frizzled from forming an active complex with these receptors, the function of Dkk3 is less clear [50], [51]. In support of our finding of *Dkk3* being upregulated by Pax6(5a) and Pax6 in the FlpIn-3T3 cell lines, *Dkk3* was downregulated in *Pax6^Sey^−/−* cortex compared to wild type mice at day E15 in a published gene expression microarray [34]. Interestingly, with regard to Pax6 role in regulation of the Wnt signaling pathway, Pax6 has recently been shown to be required for downregulation of canonical Wnt signaling in the lens ectoderm by directly controling the expression of the Wnt inhibitors *Sfrp1*, *Sfrp2* and *Dkk1* [52].

A Pax binding site in the neural cell adhesion molecule (*Ncam1*) promoter was identified as far back as in 1994 [53], and Pax6 binding to this site was subsequently shown [37]. Recently, functional assays in *Xenopus* confirmed that Pax6 directly regulates *Ncam* expression in retina [38]. These studies all identify Pax6 as the regulator of *Ncam* expression. Importantly, in our study *Ncam* was only detected as a target gene in the Pax6(5a) microarray, and this was verified by real time PCR. However, we were not able to detect binding to the *Ncam1* promoter with two different primer sets used in ChIP experiments (data not shown). If Pax6(5a) also turns out to be the major activator of Ncam expression *in vivo* in the brain and eye, then one would expect the different expression ratios and expression patterns of *Pax6* and *Pax6(5a)* to have different effects on the *Ncam* expression.

The neuronal guanine nucleotide exchange factor (*Ngef*) is expressed in certain areas of the brain [54]. Ngef (also called ephexin) interacts with EphA4 and constitutes a molecular link between Eph receptors and the cytoskeleton. This upregulates the activity of RhoA and downregulates Cdc42/Rac1 activity, as well as induces changes in cell morphology [55]. EphA4 signaling is also important for retinal axon guidance [56]. In our study *Ngef* appears to be a target gene of Pax6(5a) only, being upregulated about 12 fold compared to the control cell line. By ChIP we were also able to detect direct binding of Pax6(5a) to the *Ngef* promoter in the 3T3-Pax6(5a) cell line. Interestingly, we identified *EphB2* and *EphB4* as Pax6 specific target genes, with EphB2 being 2-fold downregulated.by Pax6 and not affected by Pax6(5a) according to qPCR results. *EphB2* is reported to be involved in differential growth cone collapse and axon retraction in retinal ganglion cells [57].

Sphingosine kinase 1 (*Sphk1*) is a lipid kinase, catalyzing the phosphorylation of sphingosine to sphingosine-1-phosphate. It is one of the genes specifically downregulated by Pax6(5a) (about 3 fold as confirmed by qPCR), but seemingly not affected by Pax6. *Sphk1* is associated with oncogenesis. In addition to promoting cellular survival, proliferation and transformation, it prevents apoptosis and stimulates angiogenesis (reviewed in [58]). It is also upregulated in several types of cancer one of them being glioblastoma, where an elevated level of Sphk1 is associated with poor prognosis [59]. Expression of Pax6 in glioblastoma multiforme (GMB) is associated with good prognosis [60], but in published papers on PAX6 and glioblastoma the authors do not distinguish between the two Pax6 isoforms. It is therefore not known whether Pax6(5a) is expressed in glioblastoma, and in what frequency compared to Pax6. This would be interesting to study further with regard to the level of *Sphk1*, since not much is known about the regulation of *Sphk1* on the transcriptional level. Even though qPCR confirmed the gene expression microarray results, bioinformatic analysis could not detect any 5aCON half-sites in the *Sphk1* promoter tested. But since *Sphk1* is registered with 17 transcripts having more than 10 different 5′ starting points (Ensembl genome browser), it is possible that we did not analyse the correct *Sphk1* promoter region.

### Common target genes

Neuroblastoma, suppression of tumorigenecity, (*Nbl1*, also named *Dan* or *NO3*) is a member of a class of secreted glycoproteins which act as inhibitors of the transforming growth factor beta and bone morphogenic protein pathway. It also acts as a tumor suppressor, indicated by the fact that human *Nbl1* is mapped to a region of chromosome 1p often deleted in neuroblastomas [61]. Further, overexpression of *Nbl1* in transformed fibroblast cell lines suppressed the transformed phenotype and reduced growth rate [62]. In line with this, *Nbl1* is found to be overexpressed in pancreatic carcinoma [63]. Normal expression pattern of *Nbl1* during embryo development includes the eye, the ear, floor plate of brain, somites and limb buds [64] as well as specific areas in the brain [65]. Even though the microarray result indicated *Nbl1* to be a Pax6(5a) specific target gene, qPCR showed that *Nbl1* transcripts were repressed about 5–6 fold by both Pax6 and Pax6(5a). Inhibition of *Nbl1 in vivo* by Pax6/Pax6(5a) would repress the inhibition of the BMP signaling pathway, keeping it active. Both the BMP and TGF beta signaling pathways are shown to be active in and important for some of the tissues where Pax6 is expressed during development (eye and brain) [66], [67].

Connective tissue growth factor (*Ctgf*) encodes a cystein-rich extracellular matrix associated protein, and was one of the genes confirmed by qPCR to be upregulated by both Pax6 (6.8 fold) and Pax6(5a) (3.8 fold). *Ctgf* is expressed in a number of different cell types, but seems to be particularly important for normal development of the skeleton since *Ctgf* null mice showed significant skeletal defects [68]. Ctgf is a major contributor of fibrotic diseases [69]. These properties do not immediately seem to be connected to Pax6. However, Pax6 is involved in corneal wound healing [70], [71], and, interestingly, Ctgf is also shown to play a role in this process, where it enhances attachment and migration [72]. Expression of *Ctgf* is reported to be influenced by cell shape altering stimuli (reviewed in [73]). By the use of ChIP we showed direct binding of Pax6 and Pax6(5a) to the *Ctgf* promoter.

### Glycosyl transferases

We have verified that seven glycosyl transferases are downregulated by Pax6 and Pax6(5a) in the 3T3 cells. It is believed that 50% of all proteins are glycosylated, especially proteins expressed on the cell surface or in the extracellular region. Changes in glycosylation pattern are often observed in various diseases and in cancer (reviewed in [74]). Some of the glycosyl transferases identified in our study (*Fut8*, *Mgat3*, *St6gal1*) modify core glycosylation structures on receptors and extracellular matrix molecules [75], [76]. In general, glycosylation is shown to play a role in intra- and intercellular trafficking, molecular and cellular matrix interactions, initiation of signal transduction and in the regulation of processes such as cell growth, migration, differentiation and tumor invasion [74], [76] Interestingly, Gcnt2 is associated with cataracts – a well known feature of mutant Pax6 phenotype [77]. Already in 1974 it was suggested that the mutant gene in small-eye mice could be involved in synthesis or processing of glycoproteins, since multiple glycoconjugate abnormalities were identified in mutant eyes [78]. Kucerova and colleagues have now shown that there are indeed alterations in the glycoconjugate signature of the Pax6 mutant cells, and that these alterations restrict the ability of the corneal epithelial cells to initiate migration in response to wounding [79].

To conclude, we have generated FlpIn-3T3 cell lines expressing only the Pax6 or Pax6(5a) isoform and this approach enabled us to identify target genes specific for each of them. It also showed that several genes are regulated by both of the two Pax6 isoforms independently. These are important observations since in most cells the two Pax6 isoforms are expressed together in specific stochiometric relationships that may change over time during development of some tissues. Future studies will be needed to show if they synergistically activate the common target gene promoters. Identification of already known Pax6 target genes and observation of a link between identified target genes and known Pax6 associated biological processes indicate that the target genes found in these 3T3 cell lines are highly relevant and can contribute to the understanding of Pax6 and Pax6(5a) roles in normal development and cancer.

## Materials and Methods

### Construction and maintenance of the FlpIn-3T3 Pax6 and Pax6(5a) cell lines

FlpIn-3T3 cell lines (Invitrogen, R761-07) were grown in DMEM high glucose supplemented with penicilin, streptomycin and 10% fetal calf serum. Zeozin (100 µg/ml) was added for the FlpIn-3T3 cells, while the FlpIn-3T3 cells constructed to express Pax6 or Pax6/5a) were grown with hygromycin (150 µg/ml). Full length mouse cDNA clones of *mPax6* and *mPax6(5a)* in the pENRTR1A vector were used as a starting point to clone *mPax6* and *mPax6(5a)* into the pEF5/FRT/V5-DEST expression vector with the Gateway Technology (Invitrogen). Lipofectamine and Plus reagent (Invitrogen) were used for transfecting the Flp-In 3T3 cells according to the manufacturers recommendations. A ratio of 9∶1 between pOG44 (Invitrogen) and the Pax6 or Pax6(5a) expression plasmid was used in the co-transfection. Transfections were done in 10 cm dishes, and transfection media (without antibiotics and serum) was replaced with media with penicillin, streptomycin and 10% fetal calf serum after 3 hours. Media containing hygromycin (150 µg/ml) was added after 20 hrs to select for stable transfectants. After 3–4 passages expression of Pax6 and Pax6(5a) was confirmed by both RT-PCR and Western blot.

### Gene expression microarray

RNA was extracted from cells by use of Trizol according to the manufacturer's instruction (Invitrogen). Three individual RNA preparations from two different cell passage numbers were used for the FlpIn-3T3 control cell line and for the FlpIn-3T3 Pax6(5a) cell line, while RNA from two different passage numbers were prepared from the FlpIn-3T3 Pax6 cell line. The RNA was shipped to the Turku Centre for Biotechnology (University of Turku, Finland) where the sample quality was validated before the samples were processed and used in microarray on a Illumina Mouse Ref-8 V1.1 chip (project 070078). In total 24613 transcripts were on the chip. The data was normalized using the Quantile normalization method, and the R package Limma was used for performing the comparisons between the groups (done by the Turku Centre for Biotechnology). Two sets of thresholds were used for filtering. List of genes obtained by threshold 1 had a false discovery rate (FDR) p-value of 0.001, a cut off for fold change (FC) at 1.7 and the logFC at 0.77. For threshold 2, (FDR) p-value was set to 0.01, FC 1.5 and logFC 0.58. The data have been submitted to the GEO repository (accession number GSE31402).

### Western Blot

Cell lysates were incubated with sample buffer for 5 minutes at 100°C and run on a 10% SDS-PAGE gel (Mighty Small, Invitrogen). Blotting was performed onto a Hybond nitrocellulose membrane (GE Healthcare) using the Mighty Small blotting system (Invitrogen). The membrane was incubated with blocking buffer (PBS buffer/0.1% Tween-20/5% non-fat dried milk). Primary and secondary antibodies were diluted in the blocking buffer. Rabbit anti-Pax6 antibody (Millipore, cat#AB2237) was used in the dilution of 1∶1200, and rabbit anti-actin (Sigma, cat#A2066) 1∶2000. Anti-rabbit HRP-conjugate (BD Pharmingen, cat#554021) 1∶2000, and anti-biotin HRP-linked antibody (Cell Signaling, cat#7075) 1∶2000. Molecular weight markers used were Prestained Protein marker, Broad range (NEB, cat#77077S) and Biotinylated protein marker (Cell Signalling, cat#7727). Western blots were developed with the Western Blotting Luminol Reagent (Santa Cruz, cat#sc-2048) and images were acquired on an Image Reader LAS-3000 Fujifilm.

### RNA extraction and RT-PCR

Total RNA was extracted from cell cultures with use of the Trizol reagent according to the manufacturer's instructions (Invitrogen). Genomic DNA was removed by DNAse treatment according to the RNeasy Micro Kit protocol (Qiagen). Transcriptor Reverse Transcriptase (Roche) was then used to generate cDNA with use of random Hexamer primers supplied by Pharmacia. 5 µg RNA was used as input in a 40 µl reaction. 2 µl cDNA was used as input in a RT-PCR reaction with primers directed against HPRT. Volume of cDNA input was adjusted before gene specific primers were used (Pax6, Gcnt1, Gcnt2, St3gal6, St6gal1, Fut8, Mgat3, Ugt1a7c), see Table S3. PCR was performed using DyNAzyme (Finnzymes), annealing temperature of 57°C/30 cycles. PCR products were run on 1% agarose gels and visualized by the use of ethidium bromide. The procedure was repeated 3 times for each primer set.

### RNA extraction and reverse transcription quantitative PCR (RT-qPCR)

Total RNA was purified using the RNeasy Plus kit from Qiagen (Hilden, Germany) according to the manufacturer's instructions. Quantity and purity of the extracted RNA was then determined with the NanoDrop spectrophotometer (Thermo Fisher Scientific, Wilmington, DE). Reverse transcription of total RNA was performed using Superscript III Reverse Transcriptase kit (Invitrogen, Carlsbad, CA) according to the manufacturer's recommendations using 150 ng random hexamer primers (Fermentas International Inc., Canada). dNTP mix was purchased from Promega (Madison, WI). 500 ng total RNA was used per 20 µl cDNA reaction.

For quantification of mRNA expression levels, a Stratagene MX3000P instrument (Stratagene, La Jolla, CA) was used. Primer pairs were designed using Primer 3 software (Whitehead Institute, Cambrigde, MA) [80] and synthesized by Invitrogen (Table S4). Primers for one of the housekeeping genes (mTfrc) were purchased directly from Qiagen (Quantitect primer assay, QT00122745). So were primers for mTgfbi (Quantitect primer assay, QT00105770).

cDNA corresponding to 25 ng RNA was amplified for 40 cycles in a 25 µl SYBR green PCR mix (Brilliant II SYBR Green QPCR master mix, Stratagene) containing 200 nM of each primer. Cycling conditions were 95°C for 10 min followed by 40 cycles at 95°C for 30 sec, 60°C for 1 min and 72°C for 30 sec. A SYBR green melting curve analysis was included to determine primer specificities and confirm the absence of primer dimers.

Duplicate PCR analyses were performed on each cDNA sample. The absence of genomic DNA and contaminations were confirmed by the inclusion of no reverse transcriptase (No RT) controls and no template controls (NTCs) respectively. The relative amount of target gene normalized to the average expression of the two reference genes *Tfrc* and *Nono* was determined using the ΔΔCq-method [81].

### ChIP

Cells of 70–80% confluence were crosslinked in freshly prepared 1% formaldehyde for 7 minutes at room temperature. The crosslinking was stopped by adding 0.125 M glycine. Cells were harvested in lysis buffer containing 1% SDS, 50 mM Tris (pH 8,0), 10 mM EDTA and protease inhibitor cocktail (cat#11836153001, Roche, Germany). Chromatin was sonicated for 20 minutes using Bioruptor UCD-200 (Diagenode, Belgium), and diluted 10× with a buffer containing 0,01% SDS, 1,1% Triton X-100, 1,2 mM EDTA, 16,7 mM Tris (pH 8,0), 167 mM NaCl and protease inhibitor cocktail. For immunoprecipitation we used 1.0 µg of either of the following antibodies: human IgG (Abcam, UK, cat#AB46540-1) or anti-Pax6 (Millipore, USA, cat#AB2237). Protein A-agarose beads were purchased from Santa Cruz, USA (cat#sc-2001), pre-blocked with a mixture of 1% BSA and sonicated salmon sperm DNA (Agilent Technologies, USA, cat#201190). After IP beads were washed 4 times. Reversal of cross-links and elution of PCR-ready DNA were identically performed on immunoprecipitated DNA-protein complexes and input chromatin samples essentially as described (Nelson 2006). Chelex-100 beads were purchased from BioRad, USA (cat#142-1253) and proteinase K from Invitrogen, USA (cat#25530-015). For PCR-based verification of *in vivo* binding, we used a ready-made DyNAzyme II PCR Master Mix (Finnzymes/Thermo Fisher, USA, cat#F508). Each PCR reaction had a total volume of 25 µl with 2,0 µl template. Sequences of primers designed by Primer3 software are shown in Table S4.

### Proliferation assay

3T3 cells (control, Pax6, Pax6(5a)) were trypsinised briefly until detached. Cells were resuspended in complete growth media and counted. Optimal cell number per well (5000) was determined in initial titration experiments. According to xCELLigence (Roche) manufacturer's instructions, cells were seeded in duplicates into E-plates 16 (Roche, cat# 05469830001) after baseline measurement. Plates were incubated for 1 hour at room temperature, and then placed into the RTCA DP instrument (Roche, cat# 05469759001) located in an incubator preserving same temperature and CO_2_ concentration as were used for routine cultivation of 3T3 cells. Cell index (arbitrary unit reflecting the cell-sensor impedance) was measured every 15 minutes during the first 4 hours for better resolution at attachment and spreading phase. Further measurements were taken every 30 minutes. Slope and doubling time calculations were performed with RTCA software 1.2 (Roche). Four independent experiments were performed.

### Migration assay

Cells were serum-starved for 24 hours, trypsinised briefly until detached, then immediately resuspended in serum-free growth media. After centrifugation media was removed, pellets resuspended in serum-free media and cell numbers counted. Optimal cell number per well (20000) was determined in an initial titration experiment. According to xCELLigence manufacturer's instructions, cells were seeded in duplicates into uncoated CIM-plates 16 (Roche, cat# 05665817001) after baseline measurement. Media below membrane contained 10% of freshly thawn fetal calf serum. Plates were incubated for 1 hour at room temperature before starting the routine measurements. Cell index was measured every 15 minutes during 24 hours (duration of the experiment was limited based on manufacturer's advice to prevent errors caused by proliferation of the cells on the outer side of the membrane). Slopes were calculated with RTCA software 1.2. Three independent experiments were performed.

### Bioinformatic analyses

For Gene ontology analyses the Database for Annotation, Visualisation and Integrated Discovery (DAVID) [40], [41] was used. We used the lists of up- or downregulated genes (threshold 1) as inputs. A list of all genes on the Illumina Mouse Ref-8 V1.1 chip was provided as reference (background). The eGOn database (http://www.genetools.microarray.ntnu.no/adb/index.php) [42] was used to compare the list (both threshold 1 and threshold 2) of upregulated genes with the list of downregulated genes.

For Pax6 binding site analyses ConSite [82] was used to look for Pax6 and Bsap (Pax5) binding sites. The Bsap bs was included because Pax5 and Pax6 have similar DNA binding preferences [6]. ConSite does not have the Pax6(5a) bs as an option. To look for Pax6(5a) binding sites in selected target gene promoters, the 5aCON halfsite (ATGYTCAKTGA) [9] was used in the Genetics Computer Group (GCG) package with the *findpatterns* option, with allowed mismatch set to 2. Similar GCG analyses were also done with the the P6CON site (ANNTTCACGCWTSANTTKMNY) [16] and the Pax6 consensus site (RNGMANTSAWGCGTRAA) [6] The input to the ConSite and GCG analyses were promoters of selected target genes, identified by use of the Ensembl genome browser by including 2000 bp upstream and 500 bp downstream of the transcriptional start site (TSS) in exon 1 of the gene of interest. The identities of the transcript (and thus the regulatory regions) used for these analyses are given in table 6 and Table S3.

For extended promoter analyses and comparison of evolutionary conserved sites, promoter areas 5000 bp upstream and 1000 bp downstream of the TSS of exon 1 (Ensembl) of selected target genes were used. The region corresponding to the promoter of the orthologous human gene/transcript was included. Some of the genes chosen for analyses had several transcripts and several different first exons. The NCBI browser (*align 2 sequences* option) was used to align the mouse and human sequences so that the correct/comparable promoters were used for downstream analyses. If found, the orthologous chicken and zebrafish promoters were also included. Location of these promoter regions in the genome is specified in table 6. The promoter sequences were analysed by use of rVISTA [83] for evolutionary conserved Pax6 binding sites.

## Supporting Information

Table S1 Pax6 regulated genes (threshold 1).
(XLS)

Table S2 Pax6(5a) regulated genes (threshold 1).
(XLS)

Table S3 Pax6(5a) consensus binding sites.
(EPS)

Table S4 Primer sets.
(XLS)
